# Experiência de famílias e terapeutas com um programa de telessaúde para crianças e adolescentes com paralisia cerebral durante a pandemia de COVID‐19

**DOI:** 10.1111/dmcn.70091

**Published:** 2025-11-16

**Authors:** Rachel Oliveira, Marisa Mancini, Priscilla Figueiredo, Katia Bueno, Andrew M. Gordon, Marina Brandão

**Affiliations:** ^1^ Programa de Pós‐Graduação em Ciências da Reabilitação Universidade Federal de Minas Gerais Belo Horizonte Brasil; ^2^ Associação Mineira de Reabilitação Belo Horizonte Brasil; ^3^ Departamento de Terapia Ocupacional Universidade Federal de Minas Gerais Belo Horizonte Brasil; ^4^ Teachers College Columbia University New York USA

## Abstract

**Objetivo:**

Compreender as percepções das famílias e terapeutas sobre a sua participação em um programa domiciliar individualizado via telessaúde implementado para crianças e adolescentes com paralisia cerebral (PC) durante a pandemia da COVID‐19 no Brasil.

**Método:**

Estudo qualitativo descritivo com 13 famílias de crianças/adolescentes com PC (Sistema de Classificação da Função Motora Grossa ‐ GMFCS níveis IV‐V) e 20 terapeutas que participaram de um programa domiciliar individualizado via telessaúde. Entrevistas semiestruturadas online foram realizadas com os participantes ao final da intervenção para compreender suas expectativas, desafios, benefícios e sugestões para serviços futuros. As entrevistas foram transcritas para realização de análise temática.

**Resultados:**

Os três temas foram: 1‐ Medo do desconhecido, 2‐ Novos caminhos, 3‐ Benefícios e perspectivas futuras. Os participantes reconheceram que o envolvimento ativo da família durante a intervenção, o estabelecimento de objetivos individualizados e a comunicação entre pais e terapeutas levaram a mudanças no envolvimento das crianças, nas rotinas das famílias e no empoderamento dos pais em relação ao processo de reabilitação dos seus filhos. Conclusão: O estabelecimento de parceria entre terapeutas e famílias, associando conhecimento técnico e experiência de vida, contribuiu para a implementação bem‐sucedida da intervenção. Ações futuras podem envolver a adoção de modelos de intervenção híbridos, focados nas necessidades específicas das famílias de crianças/adolescentes com paralisia cerebral.

## Graphical abstract

Experiências de pais de crianças com paralisia cerebral e terapeutas no programa individualizado.

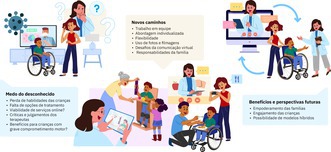

**O que este artigo acrescenta**

- Programas domiciliares individualizados são viáveis de serem implementados com ferramentas de telessaúde.
- Crianças com PC (GMFCS IV‐V) podem se beneficiar de programas domiciliares individualizados.
- A parceria entre famílias e terapeutas apoia a implementação de programas domiciliares individualizados.
- Intervenções centradas no cliente e focadas em objetivos podem ser realizadas com ferramentas de telessaúde.
- Vídeos podem ajudar famílias e terapeutas a compreenderem o desempenho das crianças.
- Os pais podem ser empoderados para implementar estratégias que facilitem as rotinas das crianças.

O isolamento social durante a pandemia da COVID‐19 dificultou o acesso presencial aos serviços de reabilitação e levou a mudanças na rotina de cuidados às crianças e adolescentes com deficiência.^1^, ^2^ O uso de ferramentas de telessaúde foi uma alternativa na prestação de serviços durante esse período.^2^, ^3^, ^4^, ^5^, ^6^ Estudos recentes reportaram os efeitos das intervenções utilizadas em conjunto com ferramentas de telessaúde durante e após a pandemia para crianças com deficiência.^1^, ^2^, ^7^, ^8^, ^9^, ^10^, ^11^, ^12^, ^13^, ^14^, ^15^, ^16^ Na maioria desses estudos, os profissionais de reabilitação selecionaram metas e atividades terapêuticas a serem realizadas pelos cuidadores.^7^, ^9^, ^13^, ^14^, ^15^ Esses estudos relataram que a maioria dos objetivos foi alcançada e as famílias relataram satisfação com a intervenção.^12^, ^13^, ^14^, ^15^ No entanto, foram destacadas limitações importantes, como desafios relacionados ao material e espaço adequados, tempo e dificuldade das famílias para compreender as atividades propostas.^13^, ^15^

Programas domiciliares são consideradas intervenções efetivas para os desfechos de funcionalidade diária de crianças com paralisia cerebral (PC).^17^, ^18^, ^19^ O programa domiciliar individualizado é uma modalidade de tratamento focada nas necessidades da família.^20^, ^21^, ^22^ Ele inclui atividades significativas que as crianças realizam em casa com a ajuda dos pais. Assim, esta intervenção respeita as preferências da família, com os terapeutas fornecendo apoio e informações, para que as famílias implementem o programa em casa, sem sobrecarga da rotina de cuidados.^20^, ^21^, ^22^, ^23^ Alguns estudos demonstraram que os programas domiciliares individualizados, utilizados em conjunto com ferramentas de telessaúde, podem efetivamente promover a funcionalidade diária de crianças com PC.^2^, ^11^, ^12^, ^16^ Os benefícios incluem a possibilidade de maior envolvimento da família, redução do stress dos cuidadores e melhora nas habilidades funcionais das crianças.^1^, ^2^, ^11^, ^12^, ^16^

Recentemente, uma meta‐síntese conduzida por Medina‐Valera et al.^19^ analisou os facilitadores e as barreiras na implementação de programas domiciliares com crianças com PC. Eles relataram que a falta de tempo para dar suporte às famílias, o fraco apoio econômico e social, bem como a situação emocional dos pais, são barreiras importantes. Os facilitadores incluíram a comunicação direta entre as famílias e os terapeutas e o uso de atividades rotineiras diárias significativas. A maioria dos programas domiciliares reportada na literatura é principalmente implementada com crianças classificadas com habilidades motoras grossas mais elevadas (i.e., classificadas como níveis I‐III no Sistema de Classificação da Função Motora Grossa‐GMFCS). Além disso, as famílias de crianças classificadas nos níveis IV e V do GMFCS têm múltiplas demandas no cuidado dos seus filhos e há pouca literatura sobre a efetividade das intervenções para essa população.^24^ O nosso estudo quantitativo anterior documentou melhora significativa nos objetivos funcionais após a conclusão de um programa domiciliar individualizado.^16^ Nesse estudo, 78,2% dos participantes eram crianças com PC classificadas nos níveis IV e V do GMFCS.^16^ O objetivo deste estudo qualitativo descritivo foi compreender as experiências das famílias de crianças classificadas nos níveis IV e V do GMFCS e dos terapeutas sobre um programa domiciliar individualizado via telessaúde implementado durante a pandemia da COVID‐19 no Brasil.

## MÉTODO

### Tipo de estudo e participantes

Um estudo qualitativo descritivo^25^ foi realizado com famílias e terapeutas que participaram de um programa domiciliar individualizado via telessaúde implementado em uma instituição de reabilitação infantil, a Associação Mineira de Reabilitação (AMR) e Universidade Federal de Minas Gerais (UFMG), durante a pandemia da COVID‐19 (agosto a dezembro de 2020).^16^ Este centro de reabilitação atende gratuitamente crianças e adolescentes com deficiências físicas e múltiplas de famílias de baixa renda que vivem na área urbana de Belo Horizonte, Brasil. A maioria das crianças/adolescentes assistidos na AMR tem PC com função motora grossa limitada. A nossa principal questão direcionou‐se a como as famílias de crianças e adolescentes com PC classificadas nos níveis IV e V da GMFCS e os terapeutas vivenciaram a implementação de um programa domiciliar individualizado via telessaúde.

Foi selecionada uma amostra por conveniência de 13 cuidadores principais (i.e., um membro da família que participou consistentemente da intervenção) de crianças e adolescentes com PC classificados nos níveis IV e V da GMFCS após a conclusão do programa domiciliar individualizado via telessaúde de 4 meses.^16^ Enviamos um e‐mail às famílias para convidá‐las para uma entrevista sobre a sua experiência no programa domiciliar. Com base no interesse e disponibilidade de cada uma das famílias, agendamos as entrevistas. As entrevistas e análises os dados foram realizadas simultaneamente para garantir que a adição de mais participantes não acrescentasse contribuições importantes sobre a questão principal do estudo, em conformidade com os princípios de saturação do conteúdo.^26^ Foram convidados^27^ terapeutas que estiveram diretamente envolvidos na implementação da intervenção para conceder uma entrevista sobre a experiência no programa domiciliar. Deste grupo, 20 terapeutas (i.e., fisioterapeutas, fonoaudiólogos, psicólogos e terapeutas ocupacionais) concordaram em participar. A Tabela 1 apresenta as informações descritivas das famílias e crianças/adolescentes, os objetivos funcionais principais reportados pela família para a intervenção e a diferença mínima clinicamente importante (DMCI) (≥2 pontos) da Medida Canadiana de Desempenho Ocupacional (COPM). A Tabela 2 apresenta as informações descritivas sobre os terapeutas.

**Tabela 1:** Informações descritivas das famílias entrevistadas e de suas crianças/adolescentes com paralisia cerebral.

Membro da família	Idade (anos)	Nível de escolaridade	Criança/adolescente	Idade (anos e meses)	Sexo	Topografia PC	GMFCS	MACS	CFCS	Objetivos funcionais	DMCI COPM Desempenho	DMCI COPM Satisfação
F1: mãe	46	Ensino fundamental incompleto	C1	16 anos e 7 meses	M	PCB	IV	III	IV	Transferência da cama para a cadeira de rodas	Sim	Sim
F2: mãe	39	Ensino superior completo	C2	5 anos e 8 meses	F	PCB	IV	IV	V	Sentar para brincar com a mãe	Sim	Sim
F3: mãe	33	Ensino fundamental completo	C3	6 anos e 5 meses	M	PCD	IV	IV	III	Usar lápis para colorir	Não	Não
F4: mãe	40	Ensino superior completo	C4	4 anos e 4 meses	F	PCB	V	V	V	Melhorar o interesse da criança em brincar	Sim	Sim
F5: mãe	34	Ensino superior completo	C5	11 anos e 3 meses	F	PCB	V	V	III	Segurar brinquedos	Sim	Sim
F6: mãe	38	Ensino superior incompleto	C6	5 anos e 9 meses	M	PCB	V	V	V	Melhorar o posicionamento da criança durante a troca de fraldas	Sim	Sim
F7: mãe	49	Ensino superior completo	C7	8 anos e 5 meses	F	PCB	IV	III	IV	Reduzir o choro quando o cabelo estiver sendo penteado	Sim	Sim
F8: mãe	34	Ensino superior completo	C8	1 ano e 2 meses	M	PCB	IV	IV	IV	Melhorar o interesse da criança em brincar	Sim	Sim
F9: pai	40	Ensino fundamental incompleto	C9	3 anos e 5 meses	F	PCB	IV	II	II	Usar uma camiseta	Sim	Sim
F10: mãe	39	Ensino médio completo	C10	5 anos e 4 meses	F	PCB	V	V	V	Melhorar a postura da criança no estabilizador ao ouvir música	Sim	Sim
F11: mãe	38	Ensino fundamental completo	C11	1 ano e 3 meses	M	PCB	V	V	V	Envolver‐se na atividade de banho	Sim	Sim
F12: mãe	39	Ensino superior completo	C12	11 anos e 8 meses	M	PCB	V	V	IV	Aumentar o tempo na postura em pé	Sim	Sim
F13: mãe	30	Ensino médio incompleto	C13	5 anos e 5 meses	F	PCB	V	V	III	Sentar para brincar com a mãe	Não	Não

F: membro da família; C: criança/adolescente; PC: paralisia cerebral; M: masculino; F: feminino; PCB: paralisia cerebral espástica bilateral; PCD: paralisia cerebral discinética; GMFCS: Sistema de Classificação da Função Motora Grossa; MACS: Sistema de Classificação da Habilidade Manual; CFCS: Sistema de Classificação da Função de Comunicação, COPM: Medida Canadense de Desempenho Ocupacional. DMCI: diferença mínima clinicamente importante nas pontuações COPM (≥2). Os dados descritivos foram obtidos a partir de registros dos prontuários após o consentimento dos participantes.

**Tabela 2:** Informações descritivas dos terapeutas entrevistados que participaram do programa domiciliar individualizado via telessaúde.

Profissionais	Idade (anos)	Sexo
TO1	55	F
TO2	46	F
TO3	36	M
TO4	31	F
TO5	51	F
TO6	37	F
TO7	41	F
FT1	30	F
FT2	28	F
FT3	30	F
FT4	46	F
FT5	40	F
PSI1	53	F
PSI2	42	F
PSI3	43	F
FGA1	40	F
FGA2	41	F
FGA3	40	F
FGA4	37	F
FGA5	43	F

**TO: terapeuta ocupacional, FT: fisioterapeuta, PSI: psicólogo; FGA: fonoaudiólogo; M: masculino; F: feminino**.

### Procedimentos

Este estudo foi aprovado pelo Comitê de Ética em Pesquisa da UFMG (CAAE: 38798520.7.0000.5149). Os participantes foram informados sobre os procedimentos e aqueles que concordaram em participar do estudo assinaram formulários de consentimento.

#### Princípios da intervenção

Os terapeutas foram previamente treinados para implementar o programa domiciliar individualizado via telessaúde, incluindo a discussão acerca de princípios de serviços centrados na família e a literatura sobre o programa domiciliar individualizado. Uma equipe interdisciplinar foi envolvida, sendo composta por fisioterapeutas, terapeutas ocupacionais, fonoaudiólogos e psicólogos. A intervenção baseou‐se no modelo proposto por Novak & Cusick.^20^ Ela consistiu em cinco fases: 1) estabelecimento de relação colaborativa com o cuidador da criança; 2) definição de objetivos funcionais com as famílias; 3) seleção das atividades terapêuticas a serem realizadas em casa; 4) suporte para implementação das atividades; 5) avaliação o progresso da criança. Resumidamente, a intervenção envolveu a seleção pela família de um objetivo funcional a ser treinado durante a rotina da criança, com o uso do COPM. Os terapeutas, em conjunto com as famílias, analisaram os vídeos do desempenho da criança no objetivo funcional escolhido (i.e., para compreender os fatores limitantes e facilitadores do desempenho da criança) e selecionaram estratégias para a prática diária da criança, incluindo o treino direto do objetivo e o uso de adaptações de baixo custo, quando apropriado. As famílias foram monitoradas pelos terapeutas semanalmente com o uso da plataforma *Google Meet* durante o período de intervenção de 4 meses (agosto a novembro de 2020). Informações sobre as especificidades da intervenção estão disponíveis em outro artigo.^16^

#### Coleta de dados

Entrevistas semiestruturadas online com participantes (i.e., cuidadores principais e terapeutas) foram realizadas imediatamente após a conclusão do programa domiciliar individualizado via telessaúde (dezembro de 2020). Os participantes foram convidados a descrever a sua rotina durante a pandemia, seguido de perguntas relacionadas com as expectativas, implementação, desafios e benefícios do programa domiciliar individualizado via telessaúde (Material Suplementar). As entrevistas foram gravadas com um gravador de voz e transcritas sem identificar os participantes (i.e., por meio de códigos), a fim de garantir a confidencialidade dos dados. As entrevistas duraram em média 35 minutos.

### Análise dos dados

Após a transcrição das entrevistas foi realizada análise temática indutiva. Este método permite a análise do conteúdo da comunicação, na qual o investigador obtém, sistematicamente, indicadores que fornecem inferências de conhecimento associadas ao fenômeno.^26^ O objetivo é compreender os pensamentos do entrevistado or meio da transcrição do conteúdo em formato de texto. Esse processo consiste em 6 etapas: 1) Pré‐análise do material, com o primeiro contato por meio de leituras flutuantes, a fim de levantar hipóteses, interpretar e organizar o material; 2) Preparação dos códigos iniciais, que identificam unidades de significado do conteúdo transcrito; 3) Criação de temas por meio de uma análise mais ampla e classificação dos códigos; 4) Revisão dos temas com refinamento das informações; 5) Definição e nomeação dos temas, que consiste em analisar a essência das particularidades de cada unidade temática; 6) Produção do texto, com refinamento dos dados significativos, interpretados para possíveis inferências e construção de argumentos dos resultados.^26^

### Confiabilidade

Os investigadores envolvidos na concepção e implementação do estudo desenvolveram um roteiro de entrevista. Anteriormente ao início da coleta de dados, pedimos a uma mãe de uma criança com PC e a um terapeuta do centro de reabilitação, que revisassem o roteiro, para garantir que o significado das perguntas fosse claro. As entrevistas foram conduzidas pela primeira autora (RO), uma terapeuta ocupacional pediátrica com experiência prévia na condução de entrevistas com famílias, mas sem contato direto prévio com os participantes. Três investigadoras com vasta experiência na condução de estudos qualitativos supervisionaram a coleta de dados e participaram ativamente na análise dos dados (KB, MC, MB). Duas investigadoras codificaram as transcrições e uma terceira investigadora triangulou posteriormente os códigos, definindo os temas. Esses temas foram apresentados e revistos em uma reunião de equipe com o envolvimento de todos os investigadores, para garantir a credibilidade e a confiabilidade dos resultados. As entrevistas foram realizadas em Português brasileiro e traduzidas para inglês após a redação dos resultados. Foram seguidas normas para publicação de estudos qualitativos Standards for Reporting Qualitative Research (SRQR).^25^

## RESULTADOS

**Figura 1:**
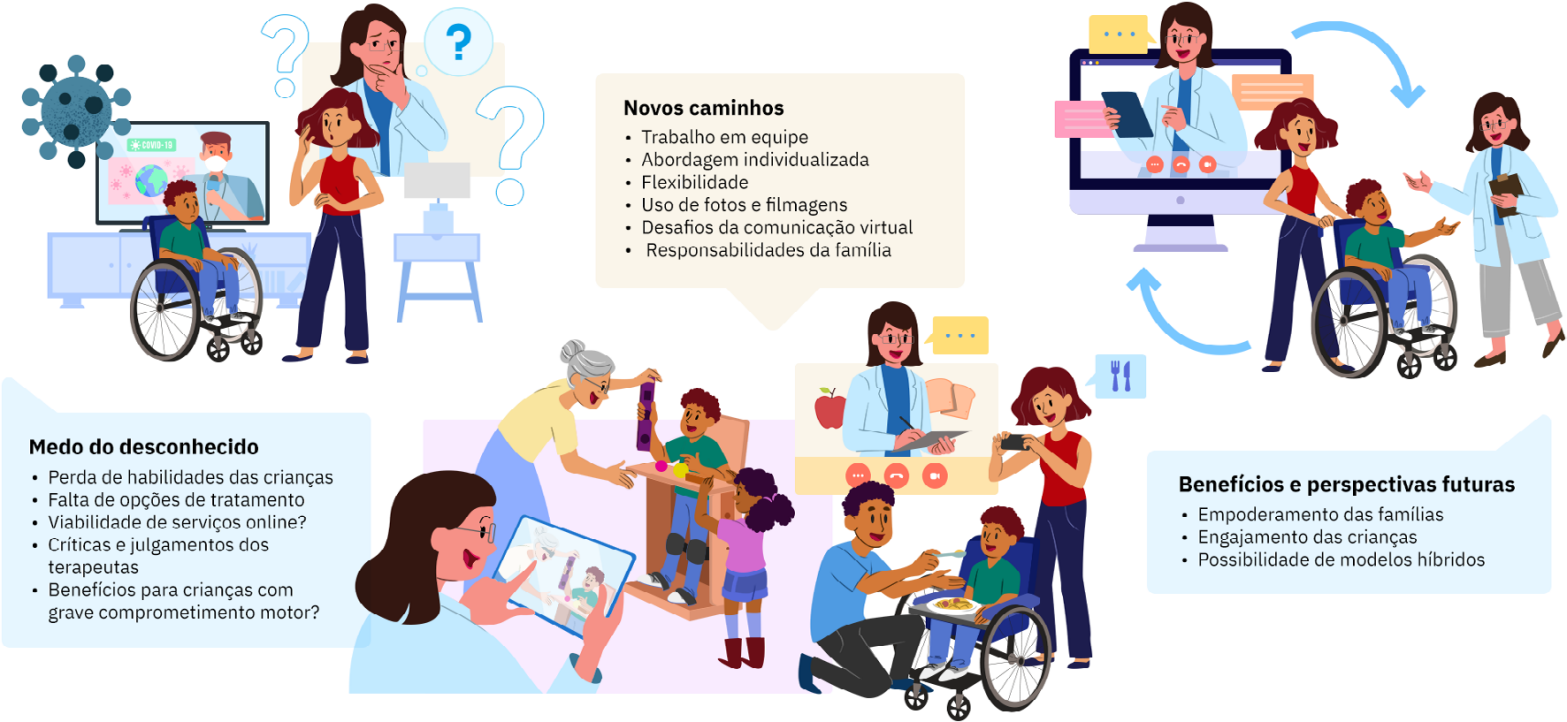
Ilustração das categorias temáticas.

Três categorias temáticas foram desenvolvidas no decorrer da análise de conteúdo das entrevistas: 1‐ Medo do desconhecido, 2‐ Vivenciando a nova proposta e 3‐ Benefícios e perspectivas futuras (Figura 1).

### Medo do desconhecido

O primeiro tema foi dividido em dois subtemas: “Perdas decorrentes do isolamento social” e “Questionamentos iniciais sobre a intervenção proposta”.

#### Perdas decorrentes do isolamento social

Tanto as famílias quanto os terapeutas expressaram preocupações com o desenvolvimento das crianças devido às restrições sociais impostas pela pandemia. As famílias estavam preocupadas com a interrupção dos serviços de reabilitação e os terapeutas relataram consequências negativas do isolamento social na participação das crianças em várias atividades.

*“Eu tinha medo de que perdêssemos a sequência do tratamento, porque ela estava acostumada a ter uma rotina de reabilitação.”* (Mãe 7)

*“Perda de experiência, habilidades motoras, porque a criança está usando menos o corpo na vida diária, está explorando menos e porque a maioria das pessoas fica presa em casa. Falta de escola, falta da experiência de sair, falta de fazer as atividades que ela costumava fazer, falta de movimento, falta de estímulo!”* (Profissional, FT2)

#### Questionamentos iniciais sobre a intervenção proposta

Inicialmente, as famílias e os terapeutas questionaram a viabilidade da intervenção proposta, pois ela não envolvia interação física entre terapeutas e crianças. As famílias expressaram preocupações sobre a possível mudança em seus papéis ao conduzir intervenções com seus filhos e questionaram se poderiam substituir os terapeutas de maneira efetiva. Os terapeutas expressaram falta de experiência com ferramentas de telessaúde e estratégias educacionais para famílias, pois suas atividades anteriores no centro de reabilitação estavam direcionadas às necessidades da criança.

*“Temos muito medo de fazer algo errado e sermos repreendidos por isso, porque você está lidando com um profissional que tem uma visão teórica completamente diferente. Como mãe, como membro da família, como cuidadora, eu faço as coisas instintivamente, porque queremos o melhor. Mas nosso instinto nem sempre corresponde às técnicas, às coisas que devem ser feitas, por isso temos muito medo de sermos criticados."* (Mãe 6).

*“No início, eu estava um pouco receosa. Telessaúde, estratégias educacionais, como isso funcionaria? Eu estava muito preocupada com isso, porque no atendimento presencial, o relacionamento geralmente se limita à criança. Como seria com a família?" (Profissional TO1)*.

Os terapeutas expressaram preocupações específicas sobre como definir objetivos com as famílias de crianças classificadas nos níveis IV e V do GMFCS. Eles questionaram se essas famílias seriam capazes de definir objetivos específicos e alcançáveis para seus filhos.

*“Como selecionar uma atividade funcional, me lembro de que estava preocupada com os mais graves. Como isso funcionaria com crianças com deficiências motoras graves? Eu tinha uma preocupação com as mães não selecionarem os objetivos corretamente, sabe? Como “brincar”, mas brincar quando, como, onde, com o quê, para que finalidade?"* (Profissional TO1).

### Novos caminhos

O segundo tema foi dividido em seis subtemas: “Estabelecimento de ações colaborativas”, “Objetivos funcionais individualizados”, “Flexibilidade na provisão de cuidados”, “Análise das atividades: compartilhamento de vídeos e soluções”, “Desafios enfrentados com a comunicação online” e “Responsabilidade da família”.

#### Estabelecimento de ações colaborativas

Os participantes destacaram as interações positivas entre os diferentes terapeutas e as famílias. Essa característica fortaleceu a relação de confiança entre terapeutas e famílias, facilitando o desenvolvimento de soluções colaborativas e a participação de outros membros da família durante a intervenção.

*“Enquanto da outra forma* [atendimento presencial], *eu levava a criança, eles* [terapeutas] *faziam o que precisava ser feito e a criança voltava para mim. Não tínhamos essas conversas, não tínhamos esses ajustes. No on‐line, foi fazer isso em família. Meu marido, por exemplo, também participou!”* (Mãe 6).

*“Conseguimos construir com a família, estabelecer metas, a família entende que ela é importante no processo, que temos uma meta funcional específica. No programa, criamos soluções. Isso é construído em conjunto, o que faz uma grande diferença!”* (Profissional, TO3)

#### Objetivos funcionais individualizados

As famílias relataram que o programa tinha um formato individualizado e que os terapeutas ofereciam apoio na seleção de objetivos funcionais específicos. Essa especificação ajudou as famílias a compreender as dificuldades de seus filhos e a importância de encontrar soluções para que eles realizassem as atividades de acordo com suas habilidades. Os terapeutas também observaram que o programa ajudou as famílias a estabelecer objetivos funcionais específicos e alcançáveis.

*“Eu sempre quis que ela sentasse sem apoio, mas eu não fazia o estímulo correto, eu estimulava ela igual estimula uma criança normal, depois com as orientações, vi que os estímulos eram de acordo com as habilidades dela”* (Mãe 5)

*“era o menino muito grave, e na hora que ela* [mãe] *viu que a demanda de transferir ele da cama, era uma coisa que ia resolver a vida dela, ela conseguiu ver isso no programa!”* (Profissional, TO1)

#### Flexibilidade na provisão de cuidados

Os participantes destacaram que a intervenção tinha um formato flexível. As famílias indicaram que o programa levou em consideração a rotina familiar. Os terapeutas reconheceram a necessidade de ajustes de acordo com as necessidades da família. Essa flexibilidade resultou em maior conhecimento sobre as habilidades e dificuldades na rotina das famílias.

*“O que pegou muito comigo é porque eu sou sozinha, né? Eu moro com meus pais, mas as coisas da M. quem faz sou eu. Mas elas* [terapeutas] *deram um tempo pra mim pra eu poder organizar meus pensamentos, é, criar mais uma rotina assim com a minha filha, sem cobrança!”* (Mãe 2)

*“Acho que nos tornamos mais flexíveis (…) Com o tempo, percebemos que o ideal é uma coisa, mas a realidade é outra. Temos de lidar com a realidade! Com o passar do tempo, tivemos mais tranquilidade para aprender sobre suas dificuldades, habilidades, facilitadores e barreiras.”* (Profissional, TO1)

#### Análise de atividades: compartilhamento de vídeos e soluções

Os terapeutas e as famílias relataram os benefícios da comunicação online. Eles destacaram a utilidade dos vídeos para a análise da atividade de forma colaborativa, com seleção de estratégias para resolver problemas e melhorar o desempenho da criança nos objetivos funcionais priorizados.

“*Quando estamos no nosso dia a dia, não vemos as coisas como estamos fazendo. Eles me pediram para gravar os vídeos e eu gravei (…) eles me deram dicas de mudanças, coisas tão simples, mas que eu não conseguia ver. Quando comecei a gravar os vídeos para mostrar a eles* [terapeutas], *eu mesma já via o vídeo, eu já via o que estava errado, eu aprendi esse hábito agora de tentar ver o problema, filmar pra eu ver a situação.”* (Mãe 7)

*“Eu acho que o fato da gente parar pra analisar os vídeos que as mães fizeram, analisar aquela atividade junto com as mães e propor junto com as mães as estratégias pra melhorar aquela atividade ajudou muito. A gente vê como que isso acontece dentro de casa junto com filmagens e fotos.”* (Profissional, FT2)

#### Desafios enfrentados na comunicação online

Os participantes relataram dificuldades em estabelecer a comunicação virtual. As famílias e os terapeutas relataram a falta de experiência com reuniões virtuais e os terapeutas expressaram desconforto em observar as rotinas das famílias durante os atendimentos virtuais.

*“Foi um pouco difícil. Porque eles estão vendo no celular, mas quando é pessoalmente, você pode ver a criança, ver o que está acontecendo. No celular, é mais difícil pra gente explicar, demonstrar e mostrar.”* (Mãe 3)

*“Assim, teve hora que eu senti que a gente era uma visita que está entrando na casa da pessoa às vezes no momento errado, sabe? Às vezes, eu sentia que estava incomodando!”* (Terapeuta, TO1)

Além disso, famílias e terapeutas destacaram dificuldades com o acesso à internet e o uso de dispositivos adequados.

*“Precisamos de um aparelho que suportasse chamadas de 40 minutos. Tivemos muitos problemas com isso.”* (Mãe 6)

*“(…) o problema da internet para as mães, no final do mês, elas não tinham internet suficiente, algumas cancelaram os atendimentos, outras não conseguiram entrar.”* (Profissional, FT4)

#### Responsabilidade da família

Famílias e os terapeutas enfatizaram a responsabilidade da família durante a implementação da intervenção. Uma mãe relatou que quase desistiu devido às dificuldades pessoais. Ela reconheceu que o apoio dos terapeutas foi essencial para a conclusão do programa.

*“Por mais que teve vez que eu tinha vontade de desistir (…) Mas são sentimentos meus, das minhas próprias dificuldades, mas a ajuda da equipe foi fundamental. Assim, sem esse teleatendimento ele não teria desenvolvido o que ele desenvolveu.”* (Mãe 8)

*“Porque se a mãe não tá ali 100% junto com a gente no teleatendimento, ele não acontece, né?”* (Profissional, TO4)

### Benefícios e perspectivas futuras

O terceiro tema foi dividido em três subtemas: “Empoderamento das famílias”, “Melhora nos objetivos funcionais da criança” e “Intervenções híbridas futuras”.

#### Empoderamento das famílias

Após a intervenção, as famílias expressaram a sensação de empoderamento em relação aos cuidados com seus filhos. Os terapeutas também relataram satisfação com o empoderamento das famílias, e essa mudança levantou questões sobre a necessidade da prestação de serviços presenciais semanalmente.

*“Hoje sou uma mãe diferente, porque consegui fazer coisas em casa, consegui mostrar a eles [terapeutas] coisas que antes não conseguia fazer com meu filho.”* (Mãe 11)

*“A gente fica feliz, da gente sair de cena e a mãe ter essa habilidade de enxergar, ter ela sempre teve, mas é dela enxergar que ela também conseguira, né? A mãe se sentindo empoderada e capaz. Mas ao mesmo tempo, a gente fica assim será que essa família precisa mesmo de mim naquele formato de toda semana?”* (Profissional, TO4)

#### Melhora da criança nos objetivos funcionais

As famílias e os terapeutas mencionaram melhora no desempenho das crianças nos objetivos priorizados, o que ocorreu com o envolvimento direto das famílias.

*“A terapeuta ocupacional me ajudou a buscar outras alternativas, um brincar que a M. gosta de fazer! Aí eu comecei a observar essas coisas, o que ela gostava de fazer e brincar com ela no que ela gostava de fazer. Ela tá até receptiva pra fazer as coisas também, sabe? Ela gosta quando é hora de brincar!*” (Mãe 2)

*“Seis meses com a criança* [antes da intervenção], *as vezes o objetivo não era alcançado, com três semanas, o mesmo objetivo foi alcançado (…) O que mais me deixou muito pensativa em relação a isso tudo, em como que o objetivo ao longo prazo era feito na clínica, e como como se tornou um objetivo de curto prazo, com o envolvimento da família!”* (Profissional, TO4)

#### Intervenções híbridas futuras

Famílias e terapeutas demonstraram interesse em continuar o programa domiciliar associado aos serviços presenciais. Ambos os grupos reforçaram a utilidade da análise das atividades com vídeos em serviços híbridos futuros.

*“Eu acho que quando voltar, não precisa nem ser pela internet, a gente gravar e mostrar pra elas e elas falarem as sugestões igual elas faziam. ”* (Mãe 7)

*“Eu não me imagino mais hoje trabalhando sem essa dinâmica de ter os vídeos das crianças. Não fico mais sem o vídeo, é sempre muito legal!”* (Profissional, FT1)

Algumas famílias relataram que a combinação de serviços online e presenciais seria útil para famílias com dificuldade em se deslocar até o centro de reabilitação. Os terapeutas também mencionaram que modelos híbridos poderiam favorecer outros membros da família a se envolverem na reabilitação da criança.

*“Eu tenho uma dificuldade em questão de deslocamento eu não moro tão próximo da AMR, eu acho que pra esse tipo de pessoa seria é esse atendimento remoto junto ao presencial seria bom.”* (Mãe 10)

*“Se a gente conseguir acessar o pai, que muitas vezes não participa, acessar uma vó que muitas vezes é quem fica com a criança e muitas vezes a equipe pode não tá ajudando ela, porque a gente não conhece as demandas e dificuldades dessa vó que tá ali no dia a dia.”* (Profissional, FT2)

## DISCUSSÃO

Nosso estudo teve como objetivo compreender as percepções das famílias e terapeutas sobre a experiência de implementação de um programa domiciliar individualizado via telessaúde com crianças e adolescentes com PC (GMFCS níveis IV‐V) durante a pandemia da COVID‐19. Os participantes passaram por uma transição para uma modalidade de atendimento na qual não tinham experiência prévia. O conteúdo que emergiu das entrevistas indicou medo inicial de mudança do tipo de serviço, a experiência de compreender os benefícios e desafios da intervenção proposta e as sugestões para implementação modelos de intervenção híbridos nos futuros serviços de reabilitação.

O envolvimento da família nos processos de reabilitação é considerado um elemento essencial na provisão de serviços centrados na família para crianças com PC.^27^ No entanto, esse é um desafio frequentemente reportado por profissionais e cuidadores de crianças com deficiência.^19^, ^28^, ^29^ As atividades do programa domiciliar individualizado via telessaúde basearam‐se no contexto natural do ambiente domiciliar, com as famílias apresentando suas prioridades e necessidades. Mais importante ainda, os participantes descreveram que o programa domiciliar possibilitou a co‐criação de soluções no ambiente doméstico e fortaleceu a relação de confiança e parceria entre terapeutas e famílias. Esses elementos contribuíram para que a família se tornasse parte integrante da equipe durante a implementação do programa domiciliar.

Em nosso estudo, pais e terapeutas consideraram a utilidade dos vídeos. A gravação das crianças realizando as atividades em casa pode facilitar os procedimentos de avaliação, pois permite a observação da criança em seu ambiente natural.^30^ Essa análise permitiu identificar barreiras e facilitadores para o desempenho das tarefas diárias das crianças no ambiente doméstico, bem como as possibilidades compartilhadas para a prática e o aprimoramento de objetivos funcionais. O uso de recursos virtuais também permite que a família se envolva no processo de intervenção com seu filho.^31^

A comunicação virtual favorece a rapidez e a fluidez da troca de informações.^1^, ^30^, ^31^ Essa agilidade também foi reportada pelos participantes do estudo. A comunicação efetiva entre famílias e profissionais é um dos facilitadores para a implementação da telessaúde,^31^ mas foram relatados desafios como a baixa qualidade da internet, a memória insuficiente dos dispositivos e a necessidade de desenvolver habilidades para usar ferramentas de comunicação. Esses achados são consistentes com estudos que descreveram os desafios da implementação de estratégias de telessaúde com crianças com deficiência, particularmente em países de baixa e média renda, durante a pandemia da COVID‐19.^15^, ^31^, ^32^, ^33^ Estudos futuros devem considerar cuidadosamente e desenvolver maneiras de minimizar possíveis desigualdades no acesso a ferramentas virtuais para pessoas com deficiência e suas famílias.

Os participantes relataram os benefícios potenciais de iniciativas futuras utilizando estratégias de telessaúde. A investigação sistemática de modelos de serviços híbridos pode ser relevante para ampliar o acesso à reabilitação para crianças e adolescentes com deficiência e suas famílias.^3^ Há evidências suficientes para sugerir que a telessaúde centrada na família é uma alternativa promissora, não apenas quando os cuidados presenciais são limitados.^1^, ^2^, ^3^, ^6^, ^11^, ^12^, ^16^ Intervenções individualizadas de telessaúde centradas na família podem aumentar a motivação dos participantes, reduzir o estresse dos cuidadores e aumentar as habilidades funcionais da criança.^6^ A combinação de serviços presenciais e virtuais também pode ser benéfica para reduzir os custos de deslocamento e o tempo de acesso aos serviços de reabilitação. Assim, a implementação de modelos híbridos pode proporcionar um acesso mais equitativo aos serviços de reabilitação, especialmente para populações de áreas remotas ou de baixa renda.^1^, ^3^, ^31^

Este estudo tem várias limitações. Os participantes relataram suas experiências após um protocolo de intervenção de quatro meses. Um período de acompanhamento mais longo poderia fornecer outros elementos para reflexão, incluindo a adesão a longo prazo a essa modalidade. Além disso, as entrevistas foram realizadas apenas com os participantes que completaram a intervenção. As famílias que desistiram não foram entrevistadas. Informações sobre essas famílias poderiam contribuir para a compreensão dos desafios enfrentados e das razões para a não adesão. Também é importante considerar que a intervenção proposta foi realizada com famílias de baixa renda. Os benefícios e desafios relatados podem ser diferentes para famílias com características socioeconômicas diferentes. Por fim, como as mães foram as principais cuidadoras que participaram do período de intervenção, a maioria das entrevistas foi realizada com elas. Portanto, é possível que as percepções de outros membros da família não tenham sido totalmente capturadas.

## CONCLUSÃO

Este estudo descreveu as experiências de famílias de crianças classificadas nos níveis IV e V do GMFCS e de seus terapeutas com um programa domiciliar individualizado via telessaúde. A participação ativa da família foi crucial para o sucesso do programa. Apesar da incerteza e do estresse da pandemia, as famílias mantiveram‐se comprometidas com as necessidades de reabilitação de seus filhos. A comunicação e a parceria entre famílias e terapeutas contribuíram para as experiências positivas relatadas. Estudos futuros que busquem compreender as percepções das equipes de reabilitação e das famílias envolvidas em modelos de intervenção híbridos focados nas necessidades da família podem favorecer a análise do impacto desse tipo de serviço. Além disso, estudos que busquem compreender como as famílias percebem a oportunidade de se envolver ativamente na reabilitação de seus filhos podem contribuir para o planejamento de intervenções focadas nas necessidades das famílias e das crianças.

### AGRADECIMENTOS

Gostaríamos de agradecer à Associação Mineira de Reabilitação, às famílias das crianças e adolescentes, aos terapeutas que participaram do estudo e a toda a equipe de reabilitação envolvida na implementação do programa. Agradecemos também aos estudantes de graduação que ajudaram na transcrição das entrevistas. The Article Processing Charge for the publication of this research was funded by the Coordenacao de Aperfeicoamento de Pessoal de Nivel Superior ‐Brasil (CAPES) (ROR identifier: 00x0ma614).

### FINANCIAMENTO

Coordenação de Aperfeiçoamento de Pessoal de Nível Superior (CAPES) (Código Financeiro 001), Conselho Nacional de Desenvolvimento Científico e Tecnológico (CNPQ).

### DECLARAÇÃO DE CONFLITO DE INTERESSES

Os autores não têm nenhum conflito de interesse a declarar.

### DECLARAÇÃO DE DISPONIBILIDADE DE DADOS

Os dados que sustentam as conclusões deste estudo estão disponíveis junto ao autor correspondente mediante solicitação razoável.

## Supporting information


**Data S1:** Supplementary Information.
